# Disheveled-1 Interacts with Claudin-5 and Contributes to Norrin-Induced Endothelial Barrier Restoration

**DOI:** 10.3390/cells12192402

**Published:** 2023-10-04

**Authors:** Mónica Díaz-Coránguez, Laura González-González, Amy Wang, Xuwen Liu, David A. Antonetti

**Affiliations:** 1 Department of Pharmacobiology, Center for Research and Advanced Studies of the National Polytechnic Institute (CINVESTAV-IPN), Mexico City 07360, Mexico; modiazco@cinvestav.mx; 2 Department of Ophthalmology and Visual Sciences, Kellogg Eye Center, University of Michigan, Ann Arbor, MI 48105, USA; gonzalla@med.umich.edu (L.G.-G.); amywang@umich.edu (A.W.); xuwen@med.umich.edu (X.L.)

**Keywords:** blood–retinal barrier, disheveled, claudin-5, tight junction, norrin, endothelium, permeability, barriergenesis, retina, Wnt signaling

## Abstract

Previous studies have revealed that norrin can reverse vascular endothelial-growth-factor (VEGF)-induced permeability in a β-catenin-dependent pathway. Here, we have explored the contribution of disheveled-1 (DVL1) in norrin-induced blood-retinal barrier (BRB) restoration. We provide evidence that in addition to canonical signaling, DVL1 promotes tight junction (TJ) stabilization through a novel, non-canonical signaling pathway involving direct claudin-5 (CLDN5) binding. Immunofluorescence staining of rat retinal cross-sections showed enriched expression of DVL1 and 3 at endothelial capillaries and co-localization with CLDN5 and ZO-1 at the TJ complex in primary bovine retinal endothelial cells (BRECs). Barrier properties of BRECs were determined via measurements of trans-endothelial electrical resistance (TEER) or permeability to 70 kDa RITC-dextran. These studies demonstrated that norrin restoration of barrier properties after VEGF treatment required DVL1 as an siRNA knockdown of Dvl1 but not Dvl2 or Dvl3, reduced basal barrier properties and ablated norrin-induced barrier restoration. However, loss of Dvl1 did not decrease β-catenin signaling activity as measured by *Axin2* mRNA expression, suggesting the contribution of a non-canonical pathway. DVL and TJ protein interactions were analyzed via co-immunoprecipitation of endogenous protein in BRECs, which demonstrated that DVL1 interacts with both CLDN5 and ZO-1, while DVL3 interacts only with ZO-1. These interactions were most abundant after inducing BRB restoration by treating BRECs with VEGF and norrin. DVL has previously been shown to form intramolecular bindings between the C-terminal PDZ-binding motif (PDZ-BM) with an internal PDZ domain. Co-transfection of HEK293 cells with DVL1 and CLDN5 or relevant mutants revealed that DVL1 interacts with CLDN5 through the DVL PDZ domain binding, CLDN5 PDZ-BM, in competition with DVL1 PDZ-BM, since DVL/CLDN5 interaction increases with deletion of the DVL1 PDZ-BM and decreases by co-expressing the C-terminal fragment of DVL1 containing the PDZ-BM or through deletion of CLDN5 PDZ-BM. In BREC cells, transfection of the C-terminal fragment of DVL1 downregulates the expression of CLDN5 but does not affect the expression of other proteins of the TJs, including ZO-1, occludin, CLDN1 or VE-cadherin. Blocking DVL1/CLDN5 interaction increased basal permeability and prevented norrin induction of barrier properties after VEGF. Combined with previous data, these results demonstrate that norrin signals through both a canonical β-catenin pathway and a non-canonical signaling pathway by which DVL1 directly binds to CLDN5 to promote barrier properties.

## 1. Introduction

Formation of the blood–retinal barrier (BRB), or barriergenesis, requires norrin, as evidenced by gene deletion studies of norrin [1,2,3,4] or any of the components of the norrin receptor complex including Frizzled 4 (FZD4) [5,6,7], low-density lipoprotein receptor-related protein 5 or 6 (LRP5/6) [8,9] and tetraspanin 12 (TSPAN12) [10,11]. Müller cells [12,13,14] and endothelial cells [15] of the developing retina express norrin that contributes to proper angiogenesis and the formation of BRB [1]. Norrin binds to the N-terminal, the extracellular, cysteine-rich domain of the FZD4 receptor and the β-propeller domains of the LRP 5/6 co-receptor [16] activating the β-catenin, canonical Wnt signaling pathway. In addition, TSPAN12 stabilizes FZD4 receptor at the cell membrane and enhances norrin-induced, but not Wnt-induced, β-catenin signaling [10].

The canonical pathway involves β-catenin stabilization and transcriptional gene regulation. In the absence of norrin or Wnt signaling, the β-catenin destruction complex formed by adenomatosis polyposis coli (APC) protein, axin, protein phosphatase 2a (PP2A), casein kinase 1α (CK1α) and glycogen synthase kinase 3 (GSK3), phosphorylates and targets β-catenin for ubiquitination and proteasomal degradation. This norrin-binding FZD4 receptor complex inactivates the APC degradation complex and inhibits GSK3 kinase, stabilizing β-catenin. Upon FZD4 receptor activation, canonical signaling is initiated through disheveled (DVL).

In mammals, there are three DVL proteins (DVL1, 2 and 3), which are between 78 and 90 kDa. DVL proteins contain a DIX domain (Dvl and axin), a PDZ domain (post-synaptic density protein-95, disc large tumor suppressor, zonula occludens-1) and a DEP domain (DVL, EGL-10, and Pleckstrin) and a C-terminal PDZ-binding motif (PDZ-BM) [17]. The activation of the canonical pathway releases DVL from FZD4, leading to DEP domain swapping between DVL molecules, initiating a head-to-tail polymerization of DVL through DIX domains [17,18]. This polymerized DVL inhibits the APC degradation complex and prevents β-catenin phosphorylation by GSK3, promoting protein stabilization. Recent studies suggest that upon ligand binding, the FZD4 receptor complex, phosphatidyl inositol [4,5] biphosphate (PIP2), increases at the plasma membrane, promoting DVL recruitment. This enhances DVL polymerization and DEP domain interaction with FZD4, followed by the inhibition of both the β-catenin destruction complex and GSK3, promoting β-catenin stabilization [19].

β-catenin stabilization and subsequent translocation to the nucleus allows interaction with the T-cell factor/lymphoid-enhancer-binding factor (TCF/LEF) complex to promote transcription (reviewed in [20]). This β-catenin-driven gene transcription is indeed required for proper vascular formation as gene deletion of β-catenin leads to fragile vessels, including malformed lumen and hemorrhagic vessels [21], and inhibition of β-catenin also leads to the loss of blood–brain barrier (BBB) properties in the remaining vessels, with CLDN3 downregulation and increased plasmalemma-vesicle-associated protein (PLVAP) expression [22]. Importantly, the genetic introduction of active β-catenin in mice with norrin loss can primarily restore barrier properties to the BBB and BRB [1]. Collectively, these studies demonstrate a requirement for β-catenin signaling in BBB and BRB formation and reveal norrin as specifically regulating BRB formation.

*Dvl* gene deletion studies, both individually and in combination, clearly demonstrate a requirement of DVL signaling in vascular development with some redundancy [23]. Mice with *Dvl1* gene deletion have structurally normal brains but display social interaction abnormalities in behaviors including whisker trimming, sleeping patterns, nest building, sensorimotor gating, huddling and recognition of social hierarchy and dominance [24,25]. However, deletion of either *Dvl2* or *Dvl3* leads to multiple phenotypes, including cardiac outflow tract abnormalities, with about half of *Dvl2^−/−^* mice dying in the perinatal period and about 87% of *Dvl3^−/−^* mice dying shortly after birth. Combined *Dvl1* and *Dvl3* deletion led to lethality by E15.5, while *Dvl2* heterozygotes plus *Dvl3* deletion was lethal by E9.5. Conversely, transgene expression of *Dvl1* or *2* can rescue *Dvl3* gene deletion phenotypes and the same effect of transgene rescue was observed for *Dvl1* or *2* gene deletion, revealing redundancy in DVL signaling [26].

DVL interacts with well over 20 proteins directly or in a complex [23]. These interactions both promote canonical signaling to β-catenin and non-canonical signaling, including planar cell polarity (PCP). Recent evidence indicates that the DVL PDZ-BM at the C-terminus can loop back and bind to the PDZ domain of DVL and contributes to the regulation of canonical versus non-canonical signaling. Solution nuclear magnetic resonance (NMR) spectroscopy and competition-binding studies have revealed that the C-terminal PDZ-BM binds with high affinity to the PDZ domain of DVL, forming a closed-loop structure that effectively activates β-catenin better than a C-terminal mutant [27]. Furthermore, in *Xenopus* embryos, the expression of Dsh (the *Xenopus* equivalent of DVL) with the deletion of the C-terminal 8 amino acids or deletion of the PDZ domain both led to a higher degree of two established PCP outcomes: convergent extension phenotype and greater JNK activation when compared to wild-type Dsh. These studies provide compelling evidence that the open structure of DVL/Dsh may promote PCP, while the closed-loop structure promotes canonical signaling.

Recently, we demonstrated that in addition to barriergenesis, norrin can reverse VEGF-induced endothelial permeability in culture and in the retinal vasculature in vivo and reverse diabetes-induced permeability [28]. VEGF was shown to induce endothelial permeability but also to promote TSPAN12 migration to the plasma membrane promoting norrin signaling and barrier restoration if norrin was provided. A role for canonical signaling through β-catenin was clearly established in this model. However, inhibition of GSK3 was not sufficient to promote barrier formation after VEGF-induced permeability even though β-catenin stabilization and signaling were observed, suggesting additional signaling pathways may contribute to norrin-induced barrier induction. Here, we provide evidence that DVL controls BRB function through canonical and non-canonical signaling pathways. Using knockdown strategies, we demonstrate that norrin signaling specifically requires DVL1 to stimulate barrier properties after VEGF-induced permeability. Further, we provide evidence for a novel, non-canonical signaling role of DVL1 in regulating barrier properties through direct binding of DVL1 PDZ domain to CLDN5 PDZ-BM. Collectively, the studies suggest that an open confirmation of DVL allows CLDN5 binding and, together with β-catenin signaling, promotes barrier formation in a novel, non-canonical pathway.

## 2. Materials and Methods

### 2.1. Primary Bovine Retinal Endothelial Cell Culture (BREC) 

For in vitro studies, primary BRECs were isolated as previously described [29] Passages 2–8 were grown until confluence at 37 °C in dishes coated with 1 μg/cm^2^ of fibronectin bovine plasma (Sigma-Aldrich, ST. Louis, MI, USA). Then, MCDB-131 media with 1% FBS and 100 nM of hydrocortisone (HC) (Sigma-Aldrich) was added to the monolayers to improve trans-endothelial electrical resistance (TEER) to over 3500 ohms. Then, cells were stimulated with the recombinant human proteins, VEGF_165_ (R&D Systems) diluted in 0.1% BSA/PBS and/or norrin (R&D Systems, Minneapolis, MN, USA) diluted in 4 mM HCl. All control monolayers were given the volume equivalent to drug or protein diluents (vehicle) used in each experiment. 

Barrier properties in BRECs were determined via measurements of TEER using the electrical-cell-substrate-impedance-sensing (ECIS) Z-theta system in 8-well chamber slides (Applied Bio Physics Inc., Troy, NY, USA) [30] or through the quantification of the flux of 10 μM of 70 kDa rhodamine B isothiocyanate-(RITC) dextran (Sigma-Aldrich; R9379) across BREC monolayers over 3.5 h. Diffusive permeability (Po) (cm/sec) was calculated as previously described [31] using the equation

*P_o_* = [(*F_L_*/Δ*t*)*V_L_*]/(*F_A_A*)

where *P_o_* is in cm/s, *F_L_* is basolateral fluorescence, *F_A_* is apical fluorescence, Δ*t* is the change in time, *A* is the surface area of the filter and *V_L_* is the volume of the basolateral chamber. 

### 2.2. BREC Transfection with siRNAs or DVL Mutants 

Primary BRECs were transfected using Amaxa Nucleofector System (Amaxa, Koeln, Germany). Next, 0.5 × 10^6^ cells were electroporated with 200 ng/mL of scramble (Scr) or siDvl sequences. Complete media were used to resuspend the cells and seed them at 0.125 × 10^6^ cells/cm^2^ on ECIS chambers, transwells, coverslips or plates coated with fibronectin. Media were changed after 24 h to MCDB-131 with 1% FBS and 100 nM of HC for two additional days before adding VEGF and/or norrin. The siRNA sequences used were GCAGAGUGAAGGAAGCAAAUU (siDvl1a), ACGUCAACUUCGAGAACAUUU (siDvl1b), CGACAUGAACUUUGAGAAUUU (siDvl2) (Dharmacon, Lafayette, CO, USA) and siDvl3 (Santa Cruz, Biotechnology, Santa Cruz, CA, USA). BRECs co-transfected with plasmids encoding DVL mutants and CLDN5 or ZO-1 were resuspended in complete media and seeded at confluency (0.25 × 10^6^ cells/cm^2^). The attached cells were washed after 3 h, and the media were replaced. Then, after 3 more hours, the media were changed to MCDB-131 with 1% FBS and 100 mM of HC to develop barrier properties. The next day, more HC was added to the wells, and 24 h after transfection, cells were collected, fixed, or stimulated with VEGF_165_ for 30 min, then norrin for 30 min. Human DVL1-WT or human DVL3-WT and its mutants were cloned in a pcDNA3.1(+)-N-HA vector, and FLAG-CLDN5 was cloned in pcDNA3.1(+)-N-DYK vector, all of them by Genscript. GFP-ZO-1 was a gift from Dr. Anuska Andejelkovic (University of Michigan). pcDNA3.1(+) empty vector (EV) was used as control. All constructs were amplified in DH5α competent cells (Thermo Fisher, Scientific, Asheville, NC, USA), purified using Plasmid Plus Maxi kit (QIAGEN, Hilden, Germany) and confirmed via sequencing.

### 2.3. HEK-293 Transfection 

HEK-293 cells were grown in DMEM high glucose supplemented with 10% FBS and antibiotics. Cells were plated at 80% confluence on 6-well plates for transfection with DVL mutants. Transfection was performed with Lipofectamine 3000 (Thermo Fisher Scientific) following the standard protocol in the reduced serum medium Opti-MEM (Thermo Fisher Scientific). Next, 2 µg of plasmid was used for each DVL mutant, 4 µg of FLAG-CLDN5 or GFP-ZO-1 plasmids and 8 µg of FLAG-CLDN5-ΔPDZBM. Then, 24 or 48 h after transfection, cells were collected for co-immunoprecipitation.

### 2.4. Co-Immunoprecipitation 

BREC or HEK-293 monolayers were collected in co-immunoprecipitation (co-IP) buffer containing 2% of non-ionic detergent NP-40. Lysates were incubated at 4 degrees for 20 min, collected via scraping then centrifuged at 13,000× *g* for 13 min. Supernatants were collected for protein quantification using BioRad protein assay. Then, 400 µg of protein was separated for co-IP using HA, FLAG or GFP tag antibodies or matching control at 1 µg of antibody per sample. Primary antibodies were incubated overnight at 4 degrees under rocking, then 60 μL of protein G agarose beads were added to each lysate for antibody capture. After 2 h, samples were centrifugated, the supernatants were discarded and the beads were washed with co-IP buffer four times. Protein was eluted from beads using 60 μL of low pH (2.8) glycine buffer for 5 min at room temperature, then NuPage sample buffer (LDS/DTT mix) was added and incubated at 70 degrees for 10 min. Supernatants were collected to be processed for Western blot analysis.

### 2.5. Western Blot (WB) 

Cells were harvested in a Triton-X-100-deoxycolate-SDS-based, or 2% of non-ionic detergent NP-40 lysis buffer, and Western blotting was carried out in NuPAGE^TM^ (Invitrogen^TM^, Thermo Fisher Scientific, Asheville, NC, USA) system as described previously [32] (81). The primary antibodies used were monoclonal mouse α-Pan-DVL (B-4) (Santa Cruz, Biotechnology, Santa Cruz, CA, USA), monoclonal rabbit α-DVL2 (30D2) (Cell Signaling Technology, Danvers, MA, USA), polyclonal rabbit α-DVL3 (Sigma Aldrich, St. Louis, MO, USA), monoclonal rat α-HA high affinity (3F10) (Roahaha Roche, Indianapolis, IN, USA), mouse monoclonal α-FLAG^®^ M2 (Sigma Aldrich), mouse monoclonal α-GFP [9F9.F9] (Abcam, Boston, MA, USA), polyclonal rabbit α-CLDN5 (Invitrogen^TM^, Thermo Fisher Scientific), monoclonal rat α-ZO-1 clone R40.76 (Millipore, Sigma-Aldrich, Burlington, MA, USA) or polyclonal rabbit α-ZO-1 (Thermo Fisher Scientific). Horseradish peroxidase-conjugated secondary antibodies, α-mouse, α-rabbit, or α-rat (GE Healthcare, Logan UT, USA), were used to detect primary antibodies via chemiluminescence. Results were analyzed using AlphaView software FluorChem^TM^ systems. 

### 2.6. Immunofluorescence Staining 

Transfected BRECs were plated on Thermanox^TM^ round plastic coverslips of 13 mm diameter (Thermo Fisher Scientific). Immunofluorescence staining was performed in confluent monolayers fixed with 1% paraformaldehyde (Electron Microscopy Sciences, Hatfield, PA, USA), permeabilized 10 min with 0.2% Triton X-100, and blocked 1 h with 2% normal goat serum (Life Technologies, Thermo Fisher Scientific; 50062Z, Carlsbad, CA, USA) and 0.1% Triton X-100. The primary antibodies used were monoclonal mouse α-Pan-DVL (B-4) (Santa Cruz, Biotechnology), monoclonal rabbit α-DVL2 (30D2) (Cell Signaling Technology), polyclonal rabbit α-DVL3 (Sigma Aldrich), monoclonal rat α-HA high affinity (3F10) (Roahaha Roche); polyclonal rabbit α-CLDN5 (Invitrogen^TM^, Thermo Fisher Scientific), monoclonal rat α-ZO-1 clone R40.76 (Millipore, Sigma-Aldrich), polyclonal rabbit α-ZO-1 (Thermo Fisher Scientific) or isolectin GS-IB4 Alexa Fluor 647 (Themo Fisher Scientific) for 2 days at 4 °C. These antibodies were detected with the secondary fluorescent antibodies, goat α-mouse, α-rabbit, or α-rat Alexa Fluor 488; goat α-rabbit or α-rat Alexa Fluor 594; goat α-rabbit or α-rat Alexa Fluor 647 (Life Technologies, Thermo Fisher Scientific); and Hoechst dye (Invitrogen^TM^, Thermo Fisher Scientific) for nuclear staining overnight at 4 °C. Samples were imaged using confocal microscopy. Co-localization between DVL, CLDN5 and ZO-1 was calculated in *xz* planes from single stacks using Mander’s correlation coefficient in ImageJ software. CLDN5 or ZO-1 cell border staining was analyzed via a semi-quantitative ranking score system on a scale of 5 categories indicating the percentage of loss in cell border staining (0–100%). Three independent observers were asked to assign a ranking score to four images per condition in a masked fashion. The results of three independent experiments were summed, and the frequency of each ranking score was calculated to determine differences between conditions. 

Whole mount retinas or cryostat sections were obtained from adult Long Evans rats. Eyes were enucleated, fixed in 4% paraformaldehyde for 30 min, and washed with PBS three times. Retinas for whole mounts were dissected under a stereotaxic microscope for their staining following the same protocol as BRECs, with Pan-DVL and CLDN5 or ZO-1 antibodies. After the staining, retinas were flat mounted into microscope slices for their analysis via confocal microscopy. For cryostat sections, eyes were dehydrated in 30% sucrose after fixation and embedded in Tissue-Tek^®^ OCT for their sectioning. Then, 10 µm slices were used for staining with IB4 and Pan-DVL, DVL2 or DVL3 antibodies. All animal experiments were conducted under the Association for Research in Vision and Ophthalmology Statement for the Use of Animals in Ophthalmic and Vision Research and the guidelines of the University Committee on Use and Care of Animals at the University of Michigan.

### 2.7. qRT-PCR 

RNA was extracted using RNeasy Plus mini kit (Qiagen Inc., Hilden, Germany; 74134). cDNA was obtained from 400–1000 ng of RNA per sample processed with Omniscript Reverse Transcription kit (Qiagen Inc.). Then, specific TaqMan^TM^ gene expression assays (Thermo Fisher Scientific) were used to detect *Dvl1*, *Dvl2* or *Axin2*. *Dvl3* was detected via non-quantitative PCR using TCTCCCAGTCCTCTCCCAAG (Fwd) and GATGGGGAC ATATGCGGGAG (Rev) primers and SybrSafe (Invitrogen) for quantification. Results were normalized to β-actin mRNA and expressed as a relative change using ∆∆Ct method.

### 2.8. Statistical Analysis 

GraphPad Prism software was used to analyze data from at least three independent experiments. Graphs represent the mean ± S.D. *p* values were calculated via *t*-test or one-way or two-way ANOVA, as indicated in each figure, and *p* ≤ 0.05 was considered significant. 

## 3. Results

### 3.1. DVL1 and 3 Are Highly Expressed in Retinal Capillaries, Co-Localizing with the Tight Junction Proteins, CLDN5 and ZO-1

**Figure 1:**
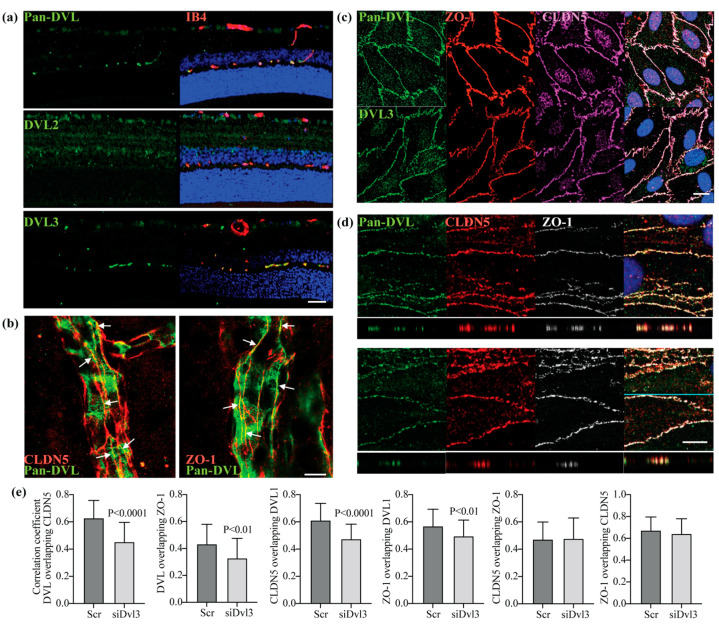
DVL1 and 3 are highly expressed in retinal capillaries, co-localizing with the TJ proteins CLDN5 and ZO-1. (**a**) Cross-sections of rat retinas immunostained with specific antibodies against DVL2 and DVL3 or with Pan-DVL antibody detecting both, DVL1 and DVL3 (green). Isolectin B4 (IB4) was used as a marker of blood-vessels (red); scale bar = 50 μM. (**b**) Co-localization of DVL (green) with the TJ proteins CLDN5 or ZO-1 (red) in rat retina whole mounts. Arrows point to the sites of co-localization in the TJs. (**c**) Co-immunofluorescence staining of DVL proteins (green), ZO-1 (red) and CLDN5 (magenta) in BREC monolayers; scale bar = 10 μM. (**d**) BRECs transfected with siDvl3 or scramble (Scr) before the IF staining with Pan-DVL (green), CLDN5 (red) and ZO-1 (white) antibodies; scale bar = 10 μM. (**e**) Protein co-localization, analyzed in xz stacks using Mander’s correlation coefficient, shows a high overlap of DVL1 with the TJ proteins in siDvl3 monolayers. *p* values were calculated via *t*-test analysis. Error bars, S.D.

Norrin restores barrier properties after VEGF-induced permeability in vivo and a primary culture of bovine retinal endothelial cells (BRECs). However, β-catenin activation by GSK3 inhibition failed to increase BREC barrier properties after VEGF despite β-catenin activation [28]. Therefore, we explored the hypothesis that DVL may signal through a non-canonical pathway to promote barrier properties in addition to β-catenin signaling. Immunostaining of cross-sections of rat retinas with Pan-DVL, DVL2 or DVL3 antibodies, along with isolectin GS-IB4 to identify retinal vessels, revealed DVL1 and 3 in the retinal capillaries (Figure 1a). Both Pan-DVL and DVL3 antibodies detected enriched expression in retinal vessels. This co-localization could be observed partially in larger arterioles but most clearly in the capillaries of the deep plexus. In contrast, DVL2 was broadly expressed across retinal tissue but with minimal vessel co-localization. Whole-mount images of retinas stained for Pan-DVL revealed localization at the cell border and co-staining with claudin-5 (CLDN5) and ZO-1 (Figure 1b).

We have confirmed the specificity of these antibodies by targeting DVL expression with siRNA in BREC cultures. Cells were grown from isolated bovine retinal capillaries as previously described [29,32]. Specific siRNA sequences were used to downregulate each DVL protein, and BREC monolayers were harvested to analyze protein expression. A representative Western blot is shown in supplementary Figure S1. Using siRNA to *Dvl2* and *Dvl3* demonstrates specific knockdown for DVL2 and DVL3 via Western blot, respectively. Pan-DVL antibody revealed a reduction of protein in the presence of siDvl1 or siDvl3, suggesting the antibody detected both forms of DVL. Unfortunately, we could not find a specific antibody for the DVL1 protein.

**Figure 2:**
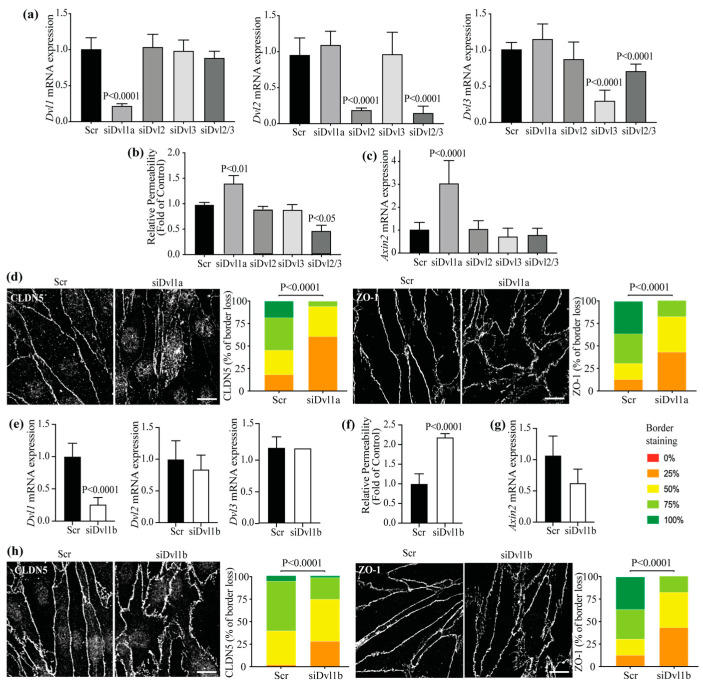
The knockdown of Dvl1 specifically, reduced basal barrier properties despite increased β-catenin signaling. (**a**) The specificity of siRNA sequences was analyzed via the qRT-PCR of Dvl1 or Dvl2 or Dvl3 PCR in BRECs transfected with siDvl1, siDvl2, siDvl3, the combined siDvl2/3 or scramble (Scr) control. Barrier properties were analyzed through the permeability to 70 kDa RITC-dextran molecule (**b**) and *Axin2* mRNA expression was measured, being accounted as β-catenin signaling activation (**c**). (**d**) Immunofluorescence staining of TJ proteins CLDN5 and ZO-1 in BREC monolayers after Dvl1 knockdown; scale bar = 10 μM. (**e**–**h**) Knockdown of Dvl1 with a second siRNA sequence. (**e**) qRT-PCR of Dvl1 or Dvl2, or Dvl3 PCR. (**f**) Solute flux. (**g**) qRT-PCR of *Axin2*. (**h**) Immunofluorescence staining of the TJ proteins; scale bar = 10 μM. *p* values were calculated via one-way ANOVA followed by Sidak post hoc test (**a**–**c**), or via *t*-test analysis (**d**–**h**). Error bars, S.D.

We further determined the localization of DVL1 and 3 in primary retinal endothelial cells. BREC monolayers were processed for the immunofluorescence staining of DVL, CLDN5 and ZO-1 proteins. Figure 1c shows enriched DVL staining at the cell contacts, co-localizing with the TJ proteins, CLDN5 and ZO-1. Because we could not find a specific antibody for DVL1 to corroborate that this protein is also expressed in retinal endothelial cells at the TJ, we knocked down *Dvl3* gene expression in BRECs using a specific siRNA. Pan-DVL antibody staining was analyzed through confocal microscopy in single z-stacks and *xz* planes. Figure 1d shows that the Pan-DVL antibody detects DVL protein enriched at the endothelial cell contacts in siDvl3 samples despite 70% *Dvl3* gene knockdown (Figure 2a). As expected, the staining was decreased in siDvl3 monolayers compared with scramble (Scr) controls due to the reduction in DVL3 content. The co-localization of this staining with CLDN5 and ZO-1 was analyzed in *xz* planes and quantified the Mander´s correlation coefficient of the pixel intensity between the two signals. Graphs in Figure 1e show over 40% correlation between Pan-DVL and TJ proteins, which was slightly decreased in the presence of siDvl3. Because siDvl3 reduced 70% of *Dvl3* gene expression (Figure 2a) and more than 80% of DVL3 protein (Figure S1), compared with Scr monolayers, we can conclude that most of the Pan-DVL staining in the siDvl3 samples indicated DVL1. Together, the data suggest that both DVL1 and 3 proteins are expressed in retinal vascular endothelial cells and co-localize at the TJ complexes.

### 3.2. Dvl1 Knockdown Reduced Basal Barrier Properties despite Increased β-Catenin Signaling

To evaluate the requirement of DVL proteins in the regulation of barrier properties, *Dvl* expression was reduced using targeted siRNA and the effect on endothelial permeability was determined. BRECs were transfected with siRNA sequences to target *Dvl 1, 2* or *3* mRNA separately. The specificity and effectiveness in knockdown of these sequences was corroborated via qRT-PCR (Figure 2a), with each siRNA inducing a specific knockdown of at least 70%. To observe the effects of *Dvl* knockdown in basal barrier properties, we performed solute flux assays on BRECs transfected with Scr control, siDvl1a, siDvl2, siDvl3 separately or siDvl2 and 3 together (siDvl2/3). Figure 2b demonstrates a 50% increase in permeability only in BRECs transfected with siDvl1a, compared with Scr monolayers. Conversely, the basal permeability was significantly reduced when siDvl2 and 3 were transfected together. This result demonstrates that DVL1 specifically promotes the barrier of retinal endothelial cells.

Because DVL proteins can activate both canonical and non-canonical signaling, we measured the mRNA expression of *Axin2*, a downstream target of β-catenin, in the presence of *Dvl* siRNAs. Surprisingly, increased *Axin2* mRNA content was found with siDvl1a (Figure 2c). That β-catenin signaling did not decrease with siDvl1 suggests that additional signaling pathways may contribute to the control of endothelial barrier properties.

Given the co-localization of DVL with the TJs, we hypothesized that *Dvl1* knockdown might alter CLDN5 and ZO-1 localization at the endothelial cell contacts. BRECs transfected with Scr or siDvl1a were processed for the immunofluorescence staining of CLDN5 and ZO-1. Using masked scoring of confocal images, we determined the percent of border localization. As shown in Figure 2d, the knockdown of *Dvl1* led to the loss of border staining for both CLDN5 and ZO-1, supporting a role for DVL1 in TJ organization.

These results were further corroborated via a second siRNA, targeting *Dvl1* (siDvl1b), which reached a similar degree of *Dvl1* knockdown compared to the first sequence (Figure 2e and Figure S2b). Consistent with our previous result, this sequence also increased basal permeability (Figure 2f) and disrupted CLDN5 and ZO-1 border localization (Figure 2h) but did not significantly alter *Axin2* mRNA expression (Figure 2g). Staining with Pan-DVL after siDvl1 knockdown revealed that the remaining DVL3 co-localized with ZO-1 both at the membrane and in the cytoplasm (S2C). Together, these results reveal that DVL1 regulates barrier properties through the regulation of TJ complex organization and suggests a non-canonical pathway that compliments β-catenin signaling.

### 3.3. Knockdown of Dvl1 but Not Dvl3, Ablated Norrin-Induced Blood–Retinal Barrier Restoration after VEGF

**Figure 3:**
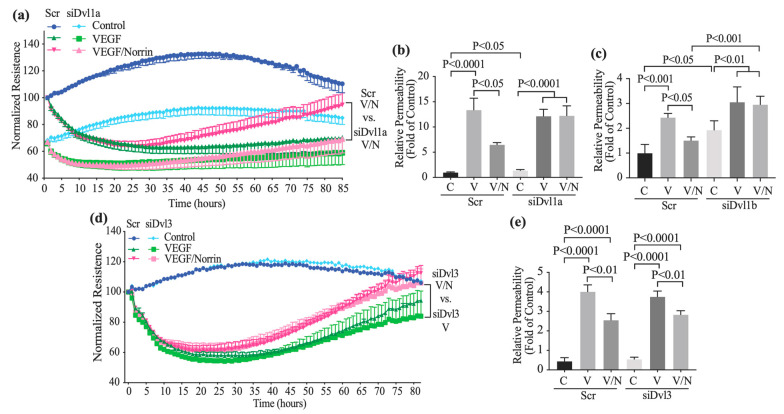
The knockdown of Dvl1 but not Dvl3-ablated norrin-induced blood–retinal barrier restoration after VEGF. (**a**,**d**) TEER or (**b**,**c**,**e**) permeability to a 70 kDa RITC-dextran molecule was measured on BRECs transfected with siDvl1a (**a**,**b**), a second siDvl1b (**c**), siDvl3 (**d**,**e**) or a scramble (Scr) control sequence. Monolayers were stimulated with vehicle (control), VEGF 50 ng/mL or VEGF and norrin 40 ng/mL (VEGF/Norrin; V/N) 72 h after transfection. Solute flux was measured after 72 h of stimulation. *p* values were calculated via two-way ANOVA, followed by Tukey´s post hoc test. Error bars, S.D.

To determine the requirement of DVL signaling in norrin-induced BRB properties after VEGF, BRECs were transfected with Scr or siDvl1 and then grown to confluence on gold-plated chambers to measure TEER using the electrical cell-substrate impedance sensing (ECIS) Z-theta system at 4000 Hz once every hour (Figure 3a). Cells were switched to media with 1% FBS and 100 nM hydrocortisone and allowed to reach a stable TEER of over 3500 ohms. In Scr monolayers, VEGF addition (50 ng/mL) induced a loss of barrier properties, as observed by a reduction in the relative TEER measure to approximately 40% of control by 24 h. The stimulation of the monolayers with norrin (40 ng/mL) alone did not affect TEER; however, co-stimulation with norrin and VEGF led first to a loss of TEER and then a complete restoration of barrier properties after 72 h as previously reported [28]. BRECs transfected with siDvl1a, however, did not reach 3500 ohms; their basal TEER stabilized at 30% lower than that of Scr monolayers. VEGF addition reduced TEER by another ~30%, and importantly, norrin failed to reverse VEGF-induced barrier disruption, revealing a required role for DVL1 in norrin-induced BRB properties.

This effect was recapitulated by testing solute flux (Figure 3b,c). BRECs were transfected with siDvl1a or scramble and were grown on 12 mm diameter, Transwell^®^ cell culture inserts, and diffusive permeability to 70 kDa dextran was determined (Po cm/sec) as previously described [31]. Scr monolayers (control, C) showed a basal permeability of 1.3 × 10^−7^ cm/s, increasing to 1.7 × 10^−6^ when the monolayers were stimulated with VEGF (Figure 3b). Co-stimulation with norrin and VEGF revealed a significant permeability reduction when compared with VEGF-stimulated monolayers. Consistent with measurements of basal TEER, siDvl1a monolayers had higher dextran permeability than Scr monolayers. VEGF increased the permeability of siDvl1a monolayers; however, norrin failed to reverse this effect after *Dvl1* knockdown. Similar results were found with a second small interfering sequence, siDvl1b (Figure 3c); in these experiments, the basal permeability started in 1.14 × 10^−6^ cm/s.

To determine the role of DVL3 in norrin-induced BRB properties, TEER and solute flux were also measured in BRECs transfected with Scr or siDvl3. As shown in Figure 3d,e, a similar VEGF and norrin response was observed with Scr control monolayers. Furthermore, in the presence of siDvl3, norrin is still able to recover TEER (Figure 3d) and to reduce VEGF-induced dextran permeability (Figure 3e) on solute flux assays, suggesting that DVL3 is not required for norrin-induced BRB properties. Together, these results suggest the requirement of DVL1 but not DVL3 in norrin-induced BRB properties after VEGF.

### 3.4. DVL1 Interacts with CLDN5 and ZO-1, While DVL3 Interacts with ZO-1

**Figure 4:**
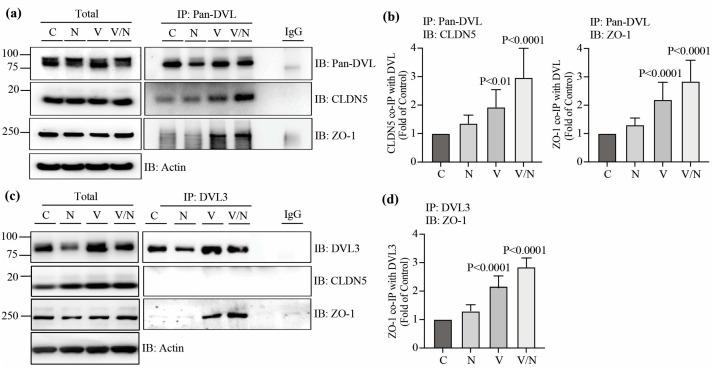
DVL1 interacts with CLDN5 and ZO-1, while DVL3 interacts with ZO-1, especially with the addition of VEGF and norrin. BREC monolayers were stimulated with vehicle (control, C), norrin 40 ng/mL (N), VEGF 50 ng/mL (V) or both (V/N). After 72 h of stimulation, lysates were collected for total protein or the immunoprecipitation (IP) of DVL using Pan-DVL (**a**,**b**) or DVL3 (**c**,**d**) specific antibodies, followed by the immunoblot (IB) with Pan-DVL, DVL3, CLDN5 or ZO-1 antibodies. Actin was used as a loading control of total protein. (**a**,**c**) Representative Western blots of total and IP protein. (**b**,**d**) Densitometry of CLDN5 or ZO-1 proteins relative to IP protein. *p* values were calculated by one-way ANOVA, followed by Sidak post hoc test. Error bars, S.D.

DVL proteins comprise DIX, PDZ and DEP domains and the relatively short PDZ-BM at the C-terminus. CLDN5 also possesses a PDZ-BM, while the ZO-1 structure contains three PDZ domains. PDZ domains are protein interaction modules that interact with other PDZ domains or PDZ-BM. Since our results demonstrate that DVL1 and 3 proteins co-localize with CLDN5 and ZO-1 in endothelial monolayers and that DVL1 is required for TJ organization and barrier properties, we tested whether DVL proteins interact directly with the TJ proteins to promote barrier function. To test this possibility, BREC monolayers were stimulated with norrin, VEGF or both for 72 h. At this time point, norrin had already begun to restore barrier properties (Figure 3). The DVL proteins were immunoprecipitated (IP), and the amount of CLDN5 and ZO-1 protein that co-IP’d with DVL was determined. As shown in Figure 4a,c, both Pan-DVL and DVL3 antibodies detected at least two bands in the total cell lysates. Based on previous literature, these are likely DVL phosphorylation sites [33] confirmed by treating with alkaline phosphatase, which collapsed all bands to one lower molecular weight (Figure S3). Norrin stimulated increased phosphorylation, as indicated via an increase in the upper molecular weight band. However, because mass spectrometry analyses have identified at least 50 serine and threonine sites of phosphorylation in DVL [34], we considered that the analysis of specific DVL phosphosites activated by norrin was beyond the scope of this manuscript. Importantly, pull-down samples denoted similar changes in DVL molecular weight, suggesting that DVL capture with these antibodies includes the phosphorylated protein.

Our results demonstrate that both CLDN5 and ZO-1 co-IP with DVL when the protein is pulled down with the Pan-DVL antibody (Figure 4a). CLDN5 had a basal interaction in non-stimulated monolayers (control, C), which was increased with the addition of VEGF (V), and even higher in the presence of both VEGF and norrin (V/N) (Figure 4b). ZO-1 had a low basal interaction with DVL; however, this was promoted with the addition of VEGF and again, the most robust interaction was induced with VEGF and norrin together. Similar changes were found by analyzing the interaction between ZO-1 and DVL3 (Figure 4c,d). However, in contrast to co-IP samples with Pan-DVL antibody, CLDN5 was not found in the co-IP of DVL3, suggesting that CLDN5 is bound to DVL1 in Pan-DVL IP samples. Together, these results indicate that ZO-1 interacts with both DVL1 and DVL3, while CLDN5 only interacts with DVL1, and these interactions are promoted by VEGF and norrin co-stimulation during the induction of BRB restoration.

Since DVL1 co-IP’d with CLDN5 and because the siRNA studies revealed DVL1 as specifically required for TJ organization at the border and norrin-induced barrier restoration, we further analyzed the structural components in DVL1 that regulate its binding to CLDN5. CLDN5 interacts with ZO-1 through a C-terminal PDZ-BM that binds to the first PDZ domain of ZO-1 [35]. Furthermore, DVL possesses both PDZ and PDZ-BM that demonstrate intramolecular binding creating a loop, as demonstrated in *Xenopus* [27,36] and by fluorescence resonance energy transfer experiments in DVL3 [37]. Thus, we tested the hypothesis that the CLDN5 PDZ-BM competes with the DVL PDZ-BM for the DVL PDZ domain. We designed a DVL1 mutant composed only of the last 169 amino acids of the C-terminus of the protein, which contains the nuclear export signals and the PDZ-BM (HA-DVL1-CT). If CLDN5 binds to the DVL1 PDZ domain, the DVL1-CT fragment would be expected to compete for this interaction. Furthermore, we designed a mutant of DVL1 lacking the PDZ-BM lacking the 7 C-terminal amino acids (HA-DVL1-ΔC7), which we hypothesized would demonstrate increased binding to CLDN5 as the PDZ domain becomes exposed.

**Figure 5:**
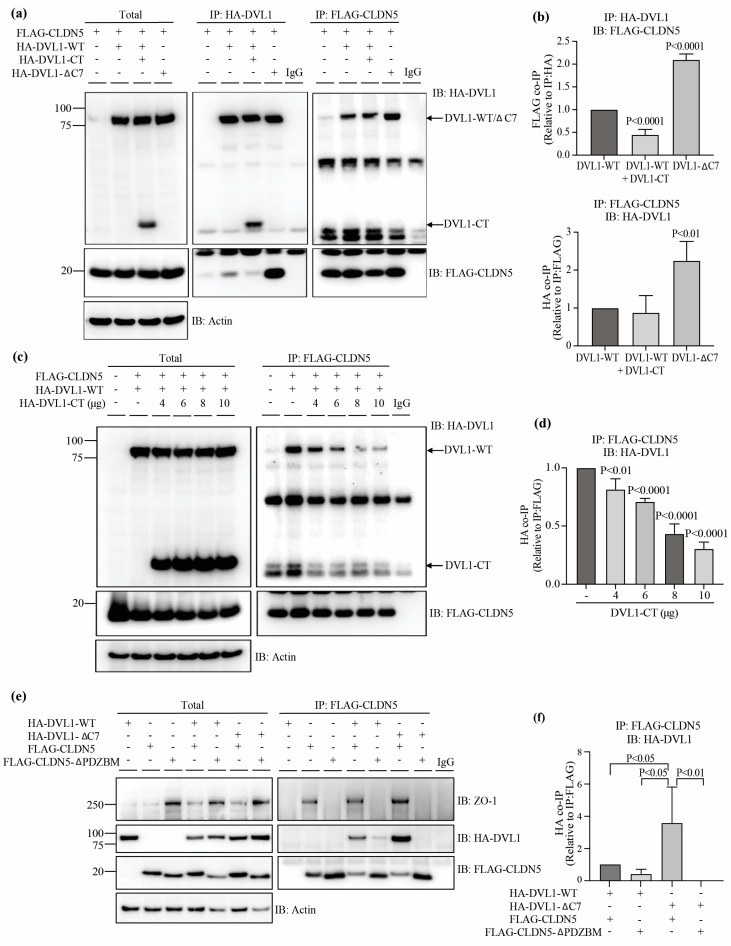
DVL1 interacts with CLDN5 through its PDZ domain. HEK-293 cells co-transfected with full-length DVL1 (HA-DVL1-WT), HA-DVL1-WT and DVL1 C-terminus (CT) 169aa fragment (HA-DVL1-CT) or Dvl1 deleted in the last 7aa which includes the PDZ-BM (HA-DVL1-ΔC7), together with FLAG-CLDN5 or FLAG-CLDN5Δ-PDZBM. Cell lysates were collected for total protein or the immunoprecipitation (IP) of DVL mutants or CLDN5, using specific antibodies against HA or FLAG tags, respectively. (**a**,**c**,**e**) Representative immunoblot (IB) of total and IP protein, using HA or FLAG antibodies. Actin was used as a loading control of total protein. (**b**,**d**,**f**) Densitometry of IP proteins. *p* values were calculated via one-way ANOVA, followed by Sidak post hoc test. Error bars, S.D.

To evaluate the possibility that CLDN5 interacts with the DVL1 PDZ domain, HEK-293 cells were co-transfected with FLAG-tagged human full-length CLDN5 (FLAG-CLDN5) and human full-length DVL1 (HA-DVL1-WT) or its mutants. Then, we performed capture experiments of the transfected mutants using either HA or FLAG tags to determine the interaction between these proteins. Transfection of FLAG-CLDN5 alone (first line in all blots) or incubation of lysates with IgG (last line in capture blots) were used as negative controls. The Western blot of the whole-cell lysates (total) showed the efficiency of protein co-transfection with similar protein content between conditions (Figure 5a). Likewise, the IP of HA-DVL (IP: HA-DVL1) or FLAG-CLDN5 (IP: FLAG-CLDN5) denoted effective pull-down of both proteins, again with similar content between samples. Pull down of HA-DVL1-WT captured FLAG-CLDN5, and this binding was reduced when co-transfected with DVL1-CT (Figure 5a,b). Further, deletion of the PDZ-BM (HA-DVL1-ΔC7) increased the interaction with FLAG-CLDN5 compared to HA-DVL1-WT. This suggests that the PDZ domain of DVL1 is required for its interaction with CLDN5 and that CLDN5 competes with the intramolecular binding from DVL-PDZ-BM. Figure 5b shows the quantification of HA-DVL1-WT/FLAG-CLDN5 co-IP blots with co-transfection of 2 µg of DVL1-CT plasmid with a significant decrease in DVL1/CLDN5 interaction when the HA tag was used for IP. However, no significant difference was observed when the FLAG tag was used for IP. To confirm that DVL1-CT competes with the CLDN5 for its interaction with DVL1-WT, HEK-293 cells were then co-transfected with FLAG-CLDN5, HA-DVL1-WT and increasing doses of HA-DVL1-CT followed by FLAG-CLDN5 IP. As shown in Figure 5c,d, 4 µg of HA-DVL1-CT plasmid was sufficient to reduce DVL1 and CLDN5 interaction significantly, and this was reduced even more as HA-DVL1-CT expression increased, thus corroborating that the C-terminus fragment of DVL1 competes with CLDN5 for the interaction to DVL1 PDZ domain.

In order to determine whether the CLDN5-PDZ-BM binds the DVL1-PDZ domain, we designed a CLDN5 lacking the carboxyl-terminal PDZBM, corresponding to the last four amino acids (FLAG-CLDN5-ΔPDZBM). FLAG-CLDN5 or FLAG-CLDN5-ΔPDZBM plasmids were transfected in HEK293 cells and co-transfected with HA-DVL1 or HA-DVL1-ΔC7 followed by FLAG-CLDN5 IP and blotting for FLAG-CLDN5, HA-DVL1 or endogenous ZO-1. Figure 5e,f shows that the IP of FLAG-CLDN5 captured HA-DVL1 and ZO-1 and that HA-DVL1-ΔC7 had increased interaction with FLAG-CLDN5. Furthermore, as expected, the deletion of the CLDN5-PDZBM impaired the binding of CLDN5 with ZO-1. The deletion of CLDN5-PDZBM reduced the interaction with HA-DVL1-WT and completely blocked binding to the HA-DVL1-ΔC7.

We also explored DVL1 or DVL3 interaction with ZO-1 and surprisingly found that each interacted with ZO-1 through distinct domains. Plasmids encoding HA-DVL1 or 3 or mutants were transfected into HEK-293 cells and co-transfected with GFP-ZO-1. Figure S4a,b shows that as with CLDN5, DVL1-WT interaction with ZO-1 was significantly reduced in the presence of DVL1-CT and increased with the deletion of DVL1 PDZ-BM, strongly implying binding to the DVL1 PDZ domain. DVL3-WT also interacted with ZO-1 (Figure S4c,d), but in contrast with DVL1, DVL3 utilizes its PDZ-BM for its interaction with ZO-1, as DVL3-ΔC7 mutant showed a shallow interaction with ZO-1. These results strongly suggest that ZO-1 can interact with DVL1 PDZ domain and DVL3 PDZ-BM.

### 3.5. DVL1 and CLDN5 Interaction Is Required for Basal and Norrin-Induced Endothelial Barrier Properties

**Figure 6:**
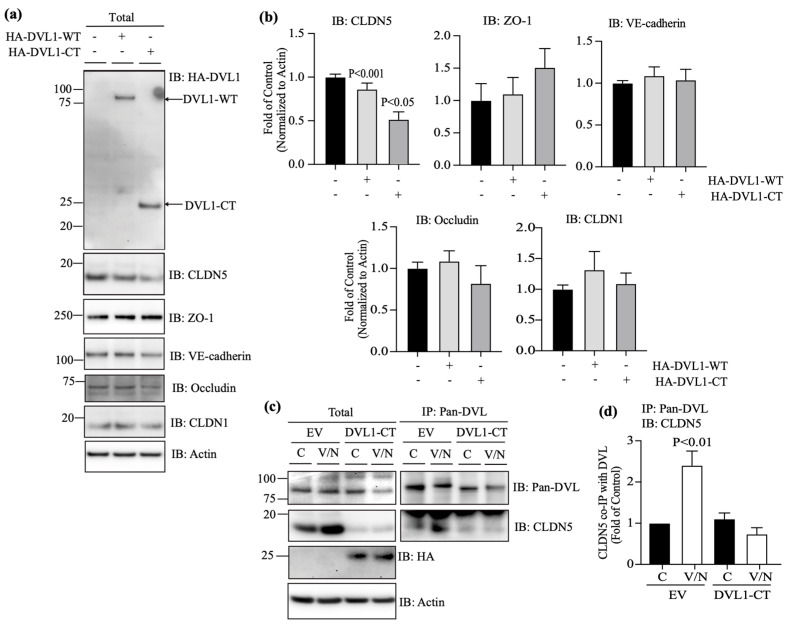
DVL1-CT fragment reduces CLDN5 content and DVL1/CLDN5 interaction in endothelial cells. BRECs were transfected with empty vector (EV), DVL1 WT (HA-DVL1) or DVL1 C-terminus (CT) 169aa fragment (HA-DVL1-CT). Some monolayers were stimulated with vehicle (control, C) or VEGF 50 ng/mL for 30 min, followed by Norrin 40 ng/mL (V/N) for additional 30 min. Cell lysates were collected for total protein or the immunoprecipitation (IP) of DVL using Pan-DVL antibody, followed by the immunoblot (IB) with Pan-DVL, CLDN5, ZO-1, VE-cadherin, occludin and CLDN1 antibodies. Actin was used as a loading control of total protein. (**a**,**c**) Representative Western blots. (**b**,**d**) Densitometry analysis. In IP experiments, CLDN5 protein was calculated relative to DVL IP protein. *p* values were calculated via one-way ANOVA, followed by Sidak post hoc test. Error bars, S.D.

To determine the role of DVL1/CLDN5 interaction in regulating endothelial barrier properties, BRECs were transfected with DVL1-CT to compete with the interaction between endogenous DVL1 and CLDN5. Measures of CLDN5 protein content when cells were transfected with the DVL1-CT revealed a dramatic loss of CLDN5 protein content (Figure 6a,b). This loss of CLDN5 was specific as CLDN1, ZO-1, occludin and VE-cadherin were not affected. Further, the immunoprecipitation of DVL1 with Pan-DVL antibody and measures of CLDN5 co-IP (Figure 6c,d) showed a dramatic loss of interaction, as expected with the loss of CLDN5 expression.

**Figure 7:**
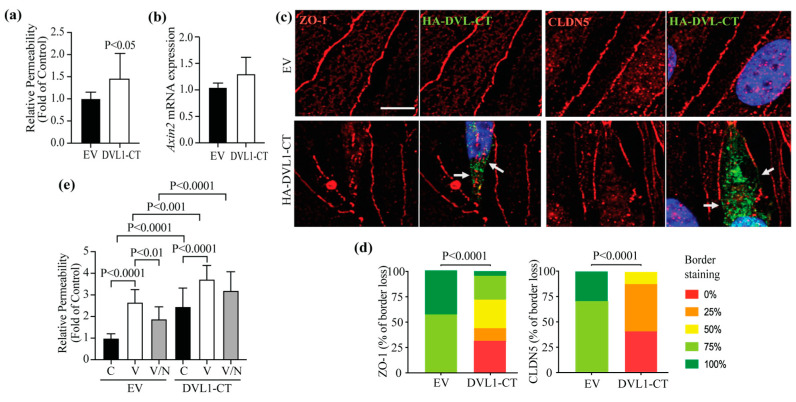
Effect of DVL1-CT in barrier properties. BRECs were transfected with empty vector (EV) or DVL1 C-terminus (CT) 169aa fragment (HA-DVL1-CT) for measurements of (**a**) basal permeability to a 70 kDa RITC-dextran molecule, (**b**) *Axin2* mRNA expression (β-catenin signaling activity) or (**c**) the IF staining of DVL1 mutants (HA-tag; green) and CLDN5 or ZO-1 TJ proteins (red); scale bar = 10 μM. Arrows point to sites of TJ protein disruption in transfected cells. (**d**) Quantification of four images per experiment by masked scoring combined from three individuals. Data show the frequency of CLDN5 or ZO-1 loss at the cell contacts ranked in five categories, where 100% corresponds to complete loss. (**e**) BREC monolayers transfected with EV or DVL1-CT fragment and stimulated with vehicle (control, C), VEGF (V) or VEGF/Norrin (V/N) for 24 h before measurements of their permeability to a 70 kDa RITC-dextran molecule. *p* values were calculated via *t*-test analysis (**a**,**b**,**d**) or via two-way ANOVA (**e**), followed by Tukey’s post hoc test. Error bars, S.D.

Consistent with the loss of CLDN5, the basal permeability of BRECs transfected with DVL1-CT increased significantly in comparison with empty vector (EV)-transfected monolayers (Figure 7a). Measurements of the β-catenin-signaling target, *Axin2*, denoted no change in canonical Wnt signaling activation in the presence of DVL1-CT (Figure 7b). As expected, the immunofluorescence staining of cells expressing DVL1-CT demonstrates that CLDN5 and ZO-1 significantly reduced endothelial border staining (Figure 7c,d). Together, these data demonstrate that DVL1/CLDN5 interaction is required to maintain basal endothelial barrier properties. To evaluate the requirement of DVL1/CLDN5 interaction in the BRB restoration induced by norrin, BREC monolayers were transfected with DVL1-CT and stimulated with VEGF or VEGF and norrin, for measurements of endothelial permeability (Figure 7e). Consistent with our previous results, in BRECs transfected with EV, VEGF increased the permeability, and norrin restored this permeability. However, when cells were transfected with DVL1-CT, the basal (control, C) permeability of these monolayers was higher than EV control monolayers. VEGF increased the endothelial permeability, but norrin failed to restore barrier properties in the presence of DVL1-CT, indicating a requirement of DVL1/CLDN5 interaction for norrin to restore barrier properties after VEGF.

**Figure 8:**
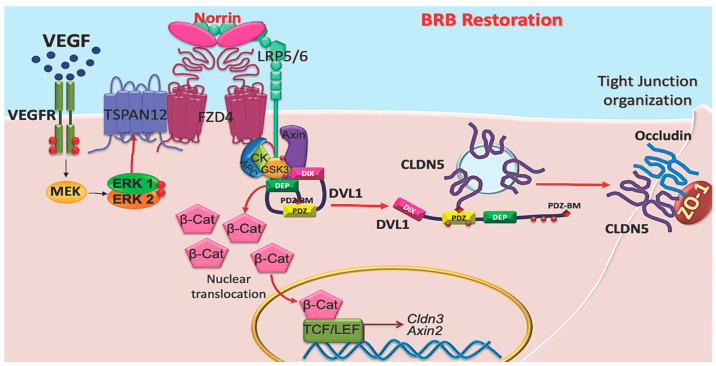
Norrin signals through DVL1 to stimulate barrier properties. Previous studies revealed that VEGF-induces endothelial permeability and simultaneously promotes norrin signaling by inducing FZD4 co-receptor TSPAN12 migration to the plasma membrane. Norrin signals through DVL1 promoting the canonical pathway with *β*-catenin stabilization, likely through the closed confirmation of DVL1 involving intramolecular binding of DVL1 PDZ domain and DVL1 PDZ-BM. Data here reveal an additional non-canonical signaling pathway necessary for tight junction organization and induction of barrier properties involving the open conformation of DVL1 with its PDZ domain binding to claudin 5 PDZ-BM.

Together, these results strongly suggest that norrin signals through DVL1 to promote BRB properties after VEGF-induced permeability. Furthermore, the studies suggest a novel, non-canonical signaling pathway activated by norrin in which DVL1/CLDN5 interaction is required to organize endothelial junctional complex and barrier function in addition to canonical β-catenin signaling (Figure 8).

## 4. Discussion

Norrin signaling through Wnt/β-catenin is required for blood–retinal barrier (BRB) development and maintenance [1]. Moreover, our previous research demonstrates that norrin can restore BRB properties after VEGF-induced permeability and that canonical signaling through β-catenin is required for this norrin effect [28]. However, data from that study also suggest that additional, non-canonical signaling may be necessary. First, *Axin2* mRNA, as a readout of β-catenin activity, did not strictly match barrier properties, as norrin alone promoted canonical signaling, and expression of *Axin2* mRNA did not affect barrier properties. In contrast, VEGF and norrin together led to *Axin2* mRNA content similar to control but promoted barrier restoration. Second, inhibition of GSK3 promoted canonical signaling but failed to promote barrier properties and, instead inhibited the ability of norrin to promote barrier properties after VEGF. Finally, the literature provides compelling evidence for the role of DVL in polarity complex formation and TJs in particular [38]. In the current study, we demonstrated that knockdown of DVL1 specifically, but not DVL2 or DVL3, reduced basal retinal endothelial barrier properties and prevented norrin restoration of barrier properties after VEGF-induced permeability without decreasing β-catenin-induced signaling. It is noteworthy that siDvl2 and 3 together actually reduced barrier properties consistent with the specific role of DVL1 in barrier promotion.

Further, inhibiting DVL1 interaction with CLDN5 by expressing the CT fragment of DVL1 containing the PDZ-BM led to the same loss of basal barrier properties and prevention of norrin restoration. The literature reveals that in development, constitutively active β-catenin can largely compensate for norrin deletion [1]. However, in vivo, polarity cues and non-canonical signaling may come from separate ligands, such as Wnts. Furthermore, the response after VEGF signaling may be different than in development. Finally, when β-catenin forced expression was used to promote the vascular barrier in regions of the brain and eye with low barrier properties, such as the circumventricular organ of the brain and choriocapillaris of the eye, the phenotypic conversion was incomplete, suggesting that additional pathways contribute to BBB and BRB formation [39].

DVL interacts with well over 20 proteins directly or in a complex [23]. These interactions both promote canonical signaling to β-catenin and non-canonical signaling, including planar cell polarity (PCP). PCP is the collective alignment of cell polarity across a tissue plain and allows for the directional pattern of tissues, for example, cilia on an apical side of an epithelial sheet [40]. PCP may be developmental or through Wnt signaling and requires FZD and DVL, among other proteins [40]. Examples of DVL signaling include the regulation of axon development in neurons with downregulation of DVL abrogating axon differentiation and overexpression of DVL, yielding multiple axons by directly binding to and activating atypical PKC (aPKC) [41]. A role for PCP in angiogenesis has been identified using pulmonary endothelial cells, where the expression of a point mutant of DVL2 that acts in a dominant negative manner to prevent PCP (K446M in the DEP domain) reduced basal cell proliferation and inhibited VEGF-induced migration. In contrast, the expression of wild-type DVL2 had no effect [42].

Furthermore, the same group demonstrated that zebrafish with a Wnt5 mutation led to vascular defects of the intersegmental vessels, dorsal aorta, posterior cardinal vein and the anterior and middle cerebral veins of the head. In this model, the Wnt5 ligand specifically activates PCP signaling and not canonical Wnt signaling. Finally, these defects could be recapitulated with a drug targeting PCP signaling. These studies reveal that DVL contributes an essential role to cell polarity, which is an essential step in TJ formation. However, none of these studies have evaluated the possibility that DVL might regulate the assembly of TJ complexes.

Here, we show for the first time a direct interaction of DVL1, specifically with CLDN5, to induce BRB assembly in both basal conditions and during norrin-induced BRB restoration. DVL1 specifically binds to CLDN5 as observed by co-IP experiments, and this interaction increases upon norrin restoration of barrier properties after VEGF treatment. Capture assays strongly suggest that the PDZ-BM of CLDN5 competes with the intramolecular binding of the PDZ-BM of DVL1 for the DVL1 PDZ domain and is consistent with previous reports suggesting the closed conformation of DVL promotes canonical signaling and the open conformation allows non-canonical signaling and in this case binding to CLDN5. Future studies must address whether this is a dynamic interaction and the relation to CLDN5 binding ZO-1. However, the current studies reveal that TJ organization requires DVL1 binding CLDN5, as both siRNA to *Dvl1* and DVL1 C-terminal fragment overexpression preventing DVL1/CLDN5 interaction disrupt TJ organization and barrier properties.

Although many reports suggest redundancy in the function of DVL proteins, here we demonstrate that in retinal endothelial cells, DVL1 has a unique contribution to BRB regulation. Specific antibodies demonstrate minimal DVL2 expression in retinal endothelial cells in the retinal cross-section, although DVL2 was observed in the cell culture. In contrast, DVL3 is enriched in retinal capillaries and shows co-localization with the TJ proteins. Nevertheless, when we knocked down *Dvl3* in endothelial cell culture with a specific siRNA, basal barrier properties did not change, and norrin could still induce BRB restoration after VEGF. However, because previous research in *Dvl3* knockout mice showed profound vascular defects, and our results also indicate that DVL3 can interact with ZO-1, particularly with VEGF and norrin stimulation in BRECs, a role for DVL3 in TJ protein regulation remains. Importantly, here we have provided for the first time, a mechanistic insight into a novel non-canonical role of DVL in the TJ biology, in which DVL1 binds directly to the TJ CLDN5 to promote endothelial barrier function. Although gene deletion of *Dvl1* in mice results in abnormal social behavior [24,25], the molecular mechanisms that lead to this phenotype and the specific role of DVL1 in BBB or BRB in vivo remain to be determined.

DVL may have additional non-canonical pathways affecting TJ. DVL binding partners, such as the PDZ-domain-containing ubiquitin ligase, PDZRN3, have been demonstrated to have a role in vascular development through PCP signaling activation. In an endothelial-specific manner, the overexpression of this ubiquitin ligase leads to the loss of cortical brain vessel barrier properties and embryonic lethality [43]. Conversely, endothelial-specific gene deletion of *Pdrzn3* protects of TJ loss and brain edema in middle-cerebral-artery-occlusion-induced stroke [43]. Cell culture studies have revealed that PDZRN3 targets the polarity protein, MUPP1, to regulate the junctional complex. PDZRN3 appears to have a central role in regulating canonical versus non-canonical signaling in endothelial cells. Using c-Jun as a marker for PCP signaling and active β-catenin as a marker for canonical signaling, endothelial-specific loss of *Pdzrn3* gene was observed to promote canonical signaling in mice [44]. This was further supported in cell culture in which depletion of *Pdzrn3* with siRNA promoted canonical signaling and inhibited c-Jun-driven non-canonical signaling. In contrast, over-expression of PDZRN3 promoted non-canonical signaling and inhibited canonical signaling, as measured via transcription reporter assays. This canonical to non-canonical signaling may be through DVL, as PDZRN3 targets DVL3 for ubiquitination [44].

DVL possesses a PDZ domain and a PDZ-BM, while ZO-1 possesses 3 PDZ domains, and these PDZ domains can bind to either C-terminal PDZ-BM, for example, allowing the binding to CLDNs through PDZ1, or can bind other PDZ domains, such as allowing ZO-ZO interaction through PDZ2 (reviewed in [45]). Here, we have found that DVL1 and DVL3 utilize different regions to interact with ZO-1; while DVL1 requires its PDZ domain, DVL3 uses its PDZ-BM. Future studies will elucidate which PDZ domain of ZO-1 is used for this interaction. It is plausible that DVL1 and 3 utilize different PDZ domains of ZO-1, and this might explain the differences in their interactions and functions.

## 5. Conclusions

Collectively, these data provide compelling evidence for a novel, non-canonical norrin signaling pathway in which DVL1 binding to claudin 5 coordinates with the canonical signaling pathway to promote the restoration of endothelial barrier properties after VEGF-induced permeability. Furthermore, this study has revealed new interactions between the PDZ-BM of CLDN5 and DVL1 necessary for tight junction organization in the vascular endothelium.

## Data Availability

Data are contained within the article or supplementary material.

## Supplementary Materials

The following supporting information can be downloaded at https://www.mdpi.com/article/10.3390/cells12192402/s1, Figure S1: Knockdown of *Dvl1*, *Dvl2*, *Dvl3* or *Dvl2/3* in BRECs using specific siRNA sequences. BREC monolayers were transfected with siRNAs targeting *Dvl1*, *2*, *3*, *2/3* or the scramble (Scr) control. Western blot analysis showing specificity of DVL2 and DVL3 antibodies and Pan-DVL antibody targeting both DVL1 and DVL3. Figure S2: Knockdown of *Dvl1* in BREC monolayers, was stable for at least 72 h. qRT-PCR of *Dvl1*, *Dvl2* or *Axin2*, or *Dvl3* PCR in BREC monolayers transfected with two specific *Dvl1* siRNA sequences: (a) first; (b) second. *p* values were calculated via *t*-test analysis. *Error bars*, S.D. (c) Immunofluorescence staining of Pan-DVL and ZO-1 in BREC monolayers after Dvl1 knockdown; scale bar = 10 μM. Figure S3: Norrin induces DVL phosphorylation. BREC monolayers were stimulated with vehicle (control, C), norrin 40 ng/mL (N), VEGF 50 ng/mL (V) or both (V/N). After 72 h of stimulation, lysates were collected. Phosphorylation was depleted using alkaline phosphatase, as shown with collapse of high-molecular-weight bands into one, confirming that DVL changes in molecular weight are due to phosphorylation. Figure S4: DVL3, but not DVL1, requires its PDZ-BM (BM) to interact with ZO-1. HEK-293 cells co-transfected with (a, b) full-length DVL1 (HA-DVL1-WT), HA-DVL1-WT and DVL1 C-terminus (CT), 169aa fragment (HA-DVL1-CT) or DVL1 deleted in the last 7aa, which includes the PDZ-BM (HA-DVL1-ΔC7), or (c, d) full-length DVL3 (HA-DVL3-WT) or DVL3 deleted in the last 7aa, which includes the PDZ-BM (HA-DVL3-ΔC7), together with GFP-ZO-1. Cell lysates were collected for total protein or the immunoprecipitation (IP) of ZO-1, using specific antibody against GFP tag. (a,c) Representative immunoblots (IB) of total and IP protein, using HA or GFP antibodies. Actin was used as a loading control of total protein. (b,d) Densitometry of IP proteins. *p* values were calculated via one-way ANOVA, followed by *Sidak* post hoc test. *Error bars*, S.D.
