# Supplementary material for: Disheveled-1 Interacts with Claudin-5 and Contributes to Norrin-Induced Endothelial Barrier Restoration
Source: Cells. 2023 Oct 4;12(19):2402. doi: 10.3390/cells12192402 (PMC10571979; doi:10.3390/cells12192402)
Supplement: Supplementary file 1 [file cells-12-02402-s001.zip › cells-2623076-supplementary.pdf]

cells MDPI

Disheveled-1 interacts with claudin-5 and contributes to norrin-induced endothelial barrier restoration

Mónica Díaz-Coránguez [1], Laura González-González [2], Amy Wang [2], Xuwen Liu [2] and David A. Antonetti [2,*]

[1] Department of Pharmacobiology, Center for Research and Advanced Studies of the National Polytechnic Institute (CINVESTAV-IPN), Mexico City, Mexico; modiazco@cinvestav.mx
[2] Department of Ophthalmology and Visual Sciences, University of Michigan, Kellogg Eye Center, Ann Arbor, MI, USA; dantonet@med.umich.edu
* Correspondence: dantonet@med.umich.edu; Tel.: 734-232-8230

Supplementary Materials:

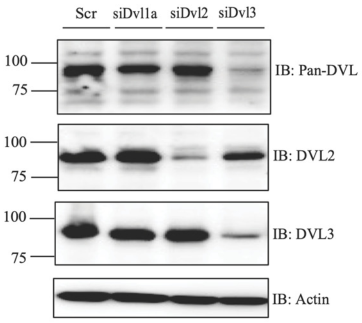

Figure S1: Knockdown of *Dvl1*, *Dvl2*, *Dvl3* or *Dvl2/3* in BREC using specific siRNA sequences. BREC monolayers were transfected with siRNAs targeting *Dvl1*, *2*, *3*, *2/3* or the scramble (Scr) control. Western blot analysis showing specificity of DVL2 and DVL3 antibodies and Pan-DVL antibody targeting both, DVL1 and DVL3.

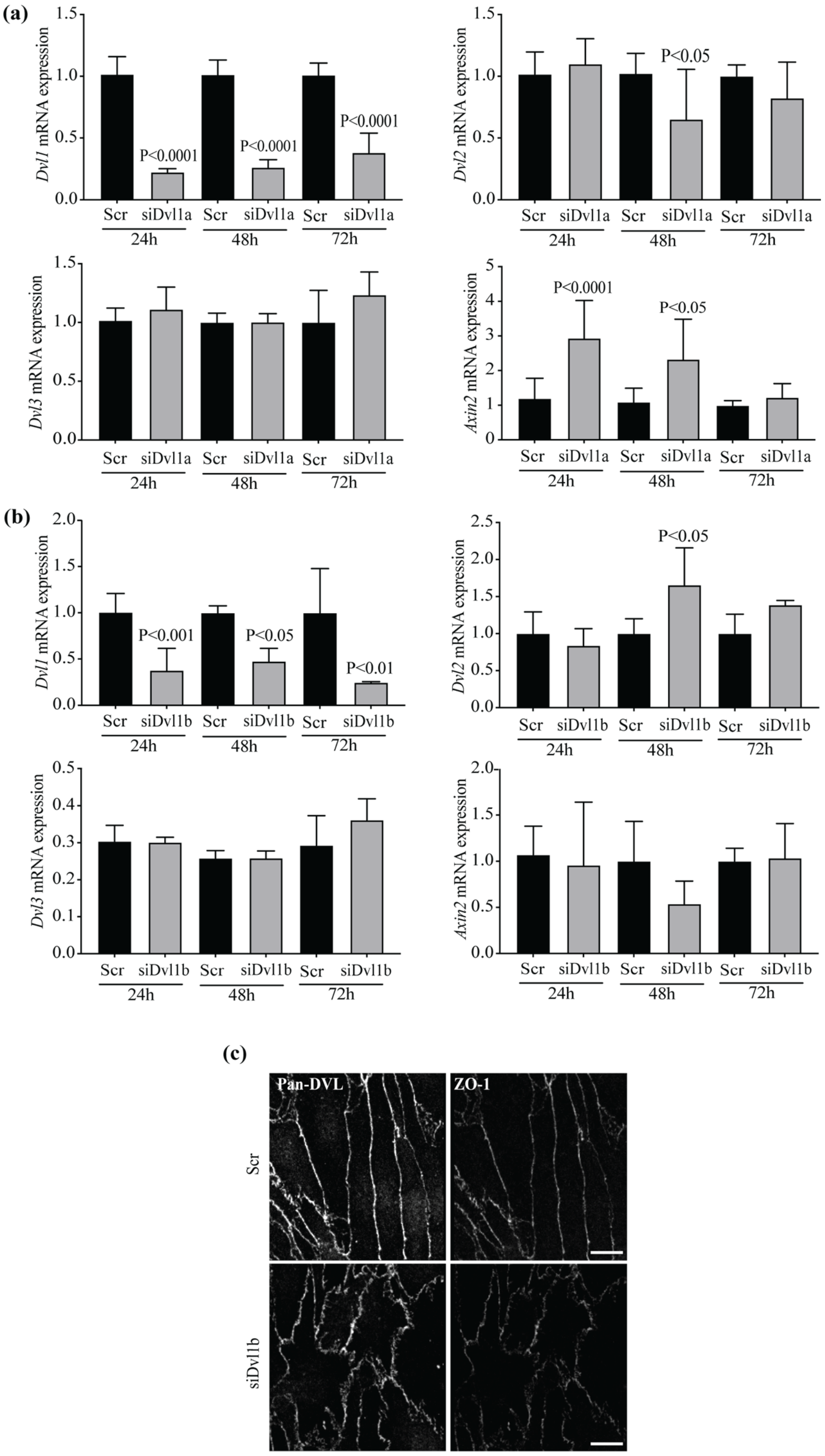

Figure S2: Knockdown of *Dvl1* in BREC monolayers, was stable for at least 72h. qRT-PCR of *Dvl1*, *Dvl2* or *Axin2*, or *Dvl3* PCR in BREC monolayers transfected with two specific *Dvl1* siRNA sequences: **(a)** first; **(b)** second. *p* values were calculated by *t-test* analysis. *Error bars*, S.D. **(c)** Immunofluorescence staining of Pan-DVL and ZO-1 in BREC monolayers after Dvl1 knockdown; scale bar= 10μM.

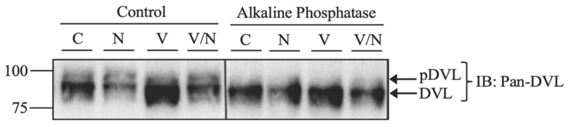

Figure S3: Norrin induces DVL phosphorylation. BREC monolayers were stimulated with vehicle (control, C), norrin 40 ng/ml (N), VEGF 50 ng/ml (V) or both (V/N). After 72h of stimulation, lysates were collected. Phosphorylation was depleted using alkaline phosphatase, as shown with collapse of high molecular weight bands into one, confirming that DVL changes in molecular weight are due to phosphorylation.

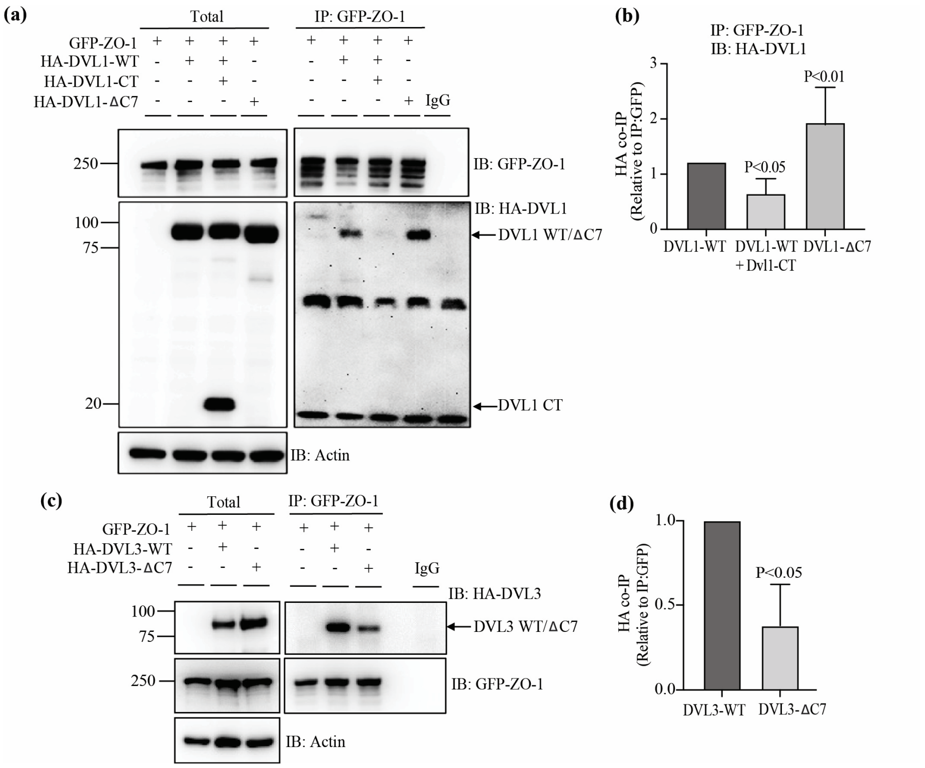

Figure S4: DVL3, but not DVL1, requires its PDZ-BM (BM) to interact with ZO-1. HEK-293 cells co-transfected with **(a, b)** full length DVL1 (HA-DVL1-WT), HA-DVL1-WT and DVL1 C-terminus (CT) 169aa fragment (HA-DVL1-CT) or DVL1 deleted in the last 7aa which includes the PDZ-BM (HA-DVL1-ΔC7), or **(c, d)** full length DVL3 (HA-DVL3-WT) or DVL3 deleted in the last 7aa which includes the PDZ-BM (HA-DVL3-ΔC7), together with GFP-ZO-1. Cell lysates were collected for total protein or the immunoprecipitation (IP) of ZO-1, using specific antibody against GFP tag. **(a, c)** Representative immunoblots (IB) of total and IP protein, using HA or GFP antibodies. Actin was used as a loading control of total protein. **(b, d)** Densitometry of IP proteins. *p* values were calculated by one-way ANOVA, followed by *Sidak* post-hoc test. *Error bars*, S.D.
